# Low-Dose Copper Exposure Exacerbates Depression-Like Behavior in ApoE4 Transgenic Mice

**DOI:** 10.1155/2021/6634181

**Published:** 2021-03-25

**Authors:** Jia Xu, Kaiwu He, Kaiqin Zhang, Chao Yang, Lulin Nie, Ding Dan, Jianjun Liu, Chang-E. Zhang, Xifei Yang

**Affiliations:** ^1^Guangzhou Municipal and Guangdong Provincial Key Laboratory of Protein Modification and Degradation, State Key Laboratory of Respiratory Disease, School of Basic Medical Sciences, Affiliated Cancer Hospital of Guangzhou Medical University, Guangdong 510000, China; ^2^Key Laboratory of Modern Toxicology of Shenzhen, Shenzhen Medical Key Subject of Modern Toxicology, Shenzhen Center for Disease Control and Prevention, Shenzhen 518055, China; ^3^School of Public Health, University of South China, Hengyang Hunan 421001, China; ^4^Cognitive Impairment Ward of Neurology Department, The Third Affiliated Hospital of Shenzhen University Medical College, Shenzhen 518001, China

## Abstract

Depression is one of the most common neuropsychiatric disorders. Although the pathogenesis of depression is still unknown, environmental risk factors and genetics are implicated. Copper (Cu), a cofactor of multiple enzymes, is involved in regulating depression-related processes. Depressed patients carrying the apolipoprotein *ε*4 allele display more severe depressive symptoms, indicating that ApoE4 is closely associated with an increased risk of depression. The study explored the effect of low-dose Cu exposure and ApoE4 on depression-like behavior of mice and further investigates the possible mechanisms. The ApoE4 mice and wild-type (WT) mice were treated with 0.13 ppm CuCl_2_ for 4 months. After the treatment, ApoE4 mice displayed obvious depression-like behavior compared with the WT mice, and Cu exposure further exacerbated the depression-like behavior of ApoE4 mice. There was no significant difference in anxiety behavior and memory behavior. Proteomic analysis revealed that the differentially expressed proteins between Cu-exposed and nonexposed ApoE4 mice were mainly involved in the Ras signaling pathway, protein export, axon guidance, serotonergic synapse, GABAergic synapse, and dopaminergic synapse. Among these differentially expressed proteins, immune response and synaptic function are highly correlated. Representative protein expression changes are quantified by western blot, showing consistent results as determined by proteomic analysis. Hippocampal astrocytes and microglia were increased in Cu-exposed ApoE4 mice, suggesting that neuroglial cells played an important role in the pathogenesis of depression. Taken together, our study demonstrated that Cu exposure exacerbates depression-like behavior of ApoE4 mice and the mechanisms may involve the dysregulation of synaptic function and immune response and overactivation of neuroinflammation.

## 1. Introduction

Depression is a common disease worldwide, with about hundreds of millions of people suffering from it [1]. With the progress of the world, the incidence of depression has been increasing year by year and its impact on society is huge. Depression can affect thoughts, mood, and physical health, and it will completely change people's understanding of the world and interpersonal relationships and even end their own lives by suicide [2]. According to the WHO analysis, depression may become the biggest burden in the world by 2030 [3, 4].

At present, it is generally believed that depression is the consequence of the interaction between genetic and environmental elements, indicating that depression is regulated by depression-related susceptible genes and environmental risk factors. Trace elements have an important role in the normal metabolism of the body and are indispensable to maintain the normal nerve system. Micronutrients are closely related to depression, and the damage of homeostasis will cause neurological dysfunction in patients. It has been found that the supplementation of trace elements can significantly improve the specific cognitive ability of patients [5–7]. Studies have found that depression is associated with trace elements, and the disruption of its homeostasis will cause neurological dysfunction in patients [8, 9]. Copper (Cu) is one of the three major trace elements, which exists in various tissues and is necessary for the body to maintain its function. Cu is an essential component of many redox enzymes in the body, and its imbalance can impair the physiological function of the nervous system [10]. As an important cofactor of neurotransmitters and signaling pathways, Cu is involved in many physiological metabolic processes of brain. Cu deficiency and overload have shown to be associated with the dysfunction of the neuropsychiatric system [11, 12]. Other studies indicated that many genes may be linked to the efficacy of depression. Since Ramachandran et al. found that the apolipoprotein E (ApoE) *ε*3/4 genotype may contribute to depressive symptoms, many researchers continuously carried out studies on the correlation between depression and the ApoE gene [13]. ApoE, a glycoprotein, played a role in the transformation and metabolism of lipoprotein and is largely expressed in the liver and brain tissue. The higher expression of ApoE is also found in glial cells of the central nervous system, suggesting the involvement of ApoE in neuroinflammation [14, 15]. ApoE gene polymorphism is related to the occurrence and development of depression, which is one of the potential risk factors for depression. It is an autosomal dominant gene with a significant genetic polymorphism, containing three alleles (*ε*2, *ε*3, and *ε*4), among which the *ε*4 allele is associated with increased risk of depression [16–18]. Notably, a meta-analysis found that ApoE4 was positively correlated with depression in people aged 23 to 83. Another community-based study also found that ApoE4 alleles were positively correlated with depression [18–20]. In addition, the presence of apolipoprotein E4 increased the risk of depression-related phenotypes [21]. Thus, these studies raise a very important question as to whether the coexistence of Cu exposure and the ApoE4 gene would aggravate the symptoms of depression and revealed its possible molecular mechanism.

In the study, we first explored the behavioral effects of low-dose Cu exposure on ApoE4 transgenic mice. The data showed that Cu exposure accelerated depression-like behavior in ApoE4 mice but had no significant effect on cognitive function. Furthermore, proteomics based on TMT labeling was employed to investigate the possible underlying mechanisms.

## 2. Material and Methods

### 2.1. Reagents

The compound copper (II) chloride (CuCl_2_) used in this research was purchased from Sigma-Aldrich, MO, USA. Urea was obtained from GE Healthcare Life Sciences (Uppsala, Sweden). DL-dithiothreitol and iodoacetamide were obtained from Sigma-Aldrich, MO, USA (St. Louis, MO, USA). Protease and phosphatase inhibitor cocktail (100×), formic acid (FA), triethylammonium bicarbonate (1 M), TMT isobaric mass tagging kit, and Pierce™ ECL Western Blotting Substrate were obtained from Thermo Fisher Scientific (Rockford, IL, USA). Sequencing grade modified trypsin was obtained from Promega, Madison (Madison, WI, USA). The antibodies used in this research included those from Cell Signaling Technology (Beverly, MA, USA), Merck KGaA (Darmstadt, Germany), Abcam (Cambridge, UK), Santa Cruz Biotechnology (Santa Cruz, CA, USA), and Invitrogen (Carlsbad, CA, USA).

### 2.2. Animals and Treatments

The experimental mice (stain: B6. Cg-ApoE^tm1Unc^Cdh18^Tg (GFAP-APOE_i4)1Hol^/J) were obtained from the Jackson Laboratory (Maine, USA). All experimental mice were housed in the Experimental Animal Center at Shenzhen Center for Disease Control and Prevention, China, and were maintained in a 12 h light-dark cycle room with stable temperature (20 ± 2°C) and humidity (55 ± 5%). Starting at 4 months of age, the mice in the treatment group were given drinking water containing 0.13 ppm Cu for 4 months [22]. After Cu exposure, the behavioral tests were carried out in the four groups (Cu-treated and untreated WT and ApoE4 mice). All animal experiments were conducted in accordance with the National Institutes of Health Guide for the Care and Use of Laboratory Animals (NIH Publications No. 8023, revised 1978). This study was authorized by the ethics committee of the experimental animal center of Shenzhen Center for Disease Control and Prevention, China.

### 2.3. Behavioral Tests

#### 2.3.1. Forced Swimming Test

Depression-like behaviors in mice were measured by the forced swimming test [23]. The test was conducted on a vertical glass cylinder (28 cm in height and 10 cm in diameter) with 20 cm of water and maintained at 23-25°C. The mice were gently placed into the water and allowed to swim freely for 6 min, and the immobility time was recorded in the last 5 min (only the head was above the water, while the body was floating in the water).

#### 2.3.2. Elevated Plus Maze Test

The elevated plus maze test is one of the most effective neuroscientific methods for assessing levels of anxious behavior in rodents [24]. The elevated plus maze test (EPM) device consists of two open arms (50 cm × 10 cm) and two closed arms (50 cm × 10 cm). The intersection is the central area (10 cm × 10 cm). At the beginning of the experiment, the mice were placed in the central platform area with their heads open arms, allowing them to explore freely. Video software was used to record the trajectory for 5 minutes and analyze the time, distance, and other indicators. After the test, wipe the maze with 75% alcohol.

#### 2.3.3. Morris Water Maze

The Morris water maze (MWM) tested the learning and memory ability of mice [25]. The test is a stainless-steel drum with a white inner wall (diameter 170 cm), in which warm water (water depth is about 30 cm) between 21°C and 22°C is injected, and an appropriate amount of skim milk powder is added to stir evenly. The platform was located in the middle of the target quadrant, 2 cm from the water surface. The mice were placed into the water from four quadrants facing the wall of the pool once, and the time of finding the platform hidden below the water surface within 60 s was recorded, which was to escape the incubation period for 5 consecutive days. After one week, the mice were tested in the pool without the platform for 2 minutes, and the escape latency and numbers of platform crossing of each mouse were calculated as the evaluation value.

### 2.4. Proteomics

#### 2.4.1. Protein Extraction and Digestion

Hippocampus tissues of mice in each group were taken and added with 8 M urea lysate. After ultrasonic lysis, they were left standing at 4°C for 30 minutes and centrifuged at 12000 rpm in a high-speed centrifuge for 30 minutes, and the supernatant was taken for use. The BCA protein assay kit (Thermo Fisher, NJ, USA) was used to measure the concentration of extracted protein. In each group, five individual samples (at a ratio of 1 : 1 : 1 : 1:1) were pooled together with a total of 100 *μ*g of proteins for enzymatic hydrolysis and labeling. Then, hippocampal protein samples were added to 10 mM DTT solution for reduction reaction for 1 h, followed by 25 mM IAA solution for 1 h at room temperature (dark environment). Each pooled protein sample was digested with trypsin for 1 h and then diluted to 1.0 M urea concentration with 1× PBS (pH 8.0), and the mixture was treated at 37°C for 15 h. After digestion, adjusting the pH to 1~2, the peptide was desalted by using a reversed-phase column (Oasis HLB, Waters, USA) and freeze-dried in a vacuum centrifuge for Tandem Mass Tag (TMT) labeling.

#### 2.4.2. TMT Labeling and LC-MS/MS Analysis

The peptides were desalinated and dried, and each sample was added with 200 mM TEAB for dissolution. Then, the peptides were labeled according to the instructions of the TMT kit. After being incubated at room temperature for 1 h, the TMT-labeled peptides were mixed, desalted, and dried and redissolved in 100 *μ*L 0.1% FA. High-performance liquid chromatography (HPLC) was used to separate TMT-labeled peptides based on specific components. Briefly, labeled peptides were loaded onto the Xbridge BEH300 C18 column (Waters, USA) for separation of peptide samples with UltiMate 3000 UHPLC (Thermo Fisher Scientific, USA) and separated into 15 fractions. Lastly, the fractions were dried and then dissolved in 20 *μ*L 0.1% FA followed by liquid chromatography- (LC-) mass spectrometry (MS)/MS analysis. The precursor ion mass tolerance was set to 20 ppm for all mass spectra obtained in the Orbitrap mass analyzer, and the fragment ion mass tolerance was corrected to 20 MMU for all MS2 spectra obtained. The row data were searched against the database of UniProt mouse FASTA with Proteome Discoverer software. The up- and downregulation of protein expression was set at ratio ≥ 1.2 and ≤0.83. All the proteomic data were deposited with the ProteomeXchange Consortium via the PRIDE partner repository with the dataset identifier PXD022422.

#### 2.4.3. Bioinformatic Analysis

WebGestalt (http://www.webgestalt.org) was used to analyze gene ontology (GO) annotation enrichment and KEGG pathway analysis (http://www.kegg.jp) of differentially expressed proteins. To explore the pattern map of Cu treatment effect, we used the R software package Mfuzz for protein cluster analysis. Heat map analysis can show the DEP abundance in different groups. The potential interaction between proteins was retrieved through STRING version 10.5 (https://string-db.org/). Lastly, Cytoscape version 3.7.2 was used for visualization of the images.

#### 2.4.4. Western Blotting

Equal amounts of protein samples were separated on 10% SDS-PAGE and transferred to PVDF membranes; then, membranes were in the blocking solution for 2 hours. The blocked membranes were incubated with antibody working solution including *α*-tubulin (1 : 3000, Merck, MAB1637), p38 MAPK (1 : 1000, Cell Signaling Technology, #9212), phospho-p38 MAPK (1 : 1000, Cell Signaling Technology, #03F9), GFAP (1 : 1000, Sigma, mab360), Iba1 (1 : 1000, Wako, 016-20001), PSD95 (1 : 1000, Abcam, ab76115), synaptophysin (1 : 1000, Abcam, ab32127), EphB2 (1 : 1000, Cell Signaling Technology, 83029T), SYN1 (1 : 1000, Abcam, ab194778), GluR1 (1 : 1000, Cell Signaling Technology, #13185), GluR2 (1 : 1000, Cell Signaling Technology, #13607), NMDA-2A (1 : 1000, Abcam, ab124913), and NMDA-2B (1 : 1000, Abcam, ab183942) overnight at 4°C. After washing in TBST buffer (3 × 10 min), membranes were in the corresponding secondary antibody and incubated for 1 h on a shaker at room temperature. Then, the membranes were exposed on phosphorimager by using an ECL kit (Thermo Scientific, NJ, USA), and the target protein analysis was performed with Quantity One (version 4.6.2) software.

#### 2.4.5. Immunohistochemistry

For immunohistochemistry, the sections were first deparaffinized in xylene and then rehydrated in gradient alcohol and then boiled for 10 min with sodium citrate (Beyotime, China) for antigen retrieval. After washing in PBS buffer (3 × 10 min), the Abcam ABC HRP Kit (ab64264) was used and all procedures were carried out according to the instructions. The sections were incubated with primary antibodies GFAP (Merck, MAB360, 1 : 250) and Iba1 (Wako Chemicals, 019 19741, 1 : 200) at 4°C overnight. After incubation, the sections were washed in PBS which was performed according to the ABC-IHC Kit manufacturer's protocol. Then, brain slices were stained for 2 min using diaminobenzidine (DAB). Finally, the sections were stained with hematoxylin, dehydrated with ethanol and xylene, and covered with coverslips. Sections were imaged by using a Leica microscope and analyzed with Image-Pro plus 6.2 software.

#### 2.4.6. ELISA of IL-10, IL-6, and TNF-*α*

The mouse brains were homogenized with 8 M urea lysate, and the lysate was left standing at 4°C for 30 minutes and centrifuged at 12000 rpm in a high-speed centrifuge for 30 minutes, and the supernatant was taken for use. The BCA protein assay kit (Thermo Fisher, NJ, USA) was used to measure the concentration of extracted proteins. The supernatant was analyzed by using mouse IL-10, IL-6, and TNF-*α* ELISA kits according to the manufacturer's instructions (E-EL-M0046c, E-EL-M0044c, and E-EL-M0049c, respectively, Elabscience). The concentrations of IL-10, IL-6, and TNF-*α* were determined by comparison with the standard curve.

#### 2.4.7. Statistical Analysis

GraphPad Prism 7.0 statistical software (La Jolla, CA, USA) was used for all statistical analyses. The data were expressed as the mean ± SEM, and statistical analysis was carried out using one-way ANOVA. *p* < 0.05 was considered to indicate statistically significant difference.

## 3. Results

### 3.1. Cu Exposure Aggravated Depression-Like Behavior in ApoE4 Mice

**Figure 1:**
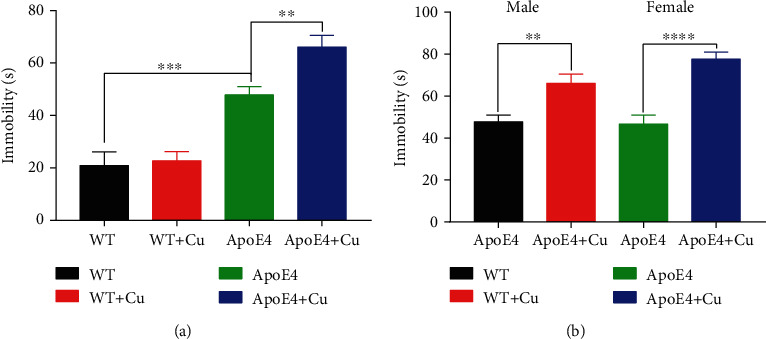
The 4 months of low-dose copper exposure on depression-like behavior in mice. (a, b) The percentage of immobility time. The data was shown as mean ± SEM. ^∗∗^*p* < 0.01, ^∗∗∗^*p* < 0.001, and ^∗∗∗∗^*p* < 0.0001. *n* = 10-12 for each group.

The forced swimming test showed that ApoE4 mice had more immobility time than WT mice, while Cu-treated ApoE4 mice spent longer immobility time than the ApoE4 mice (Figure 1(a)). Besides, the immobility time of Cu-treated ApoE4 mice was significantly longer than that of ApoE4 mice (Figure 1(b)), regardless of gender. The data suggested that Cu exposure aggravated depression-like behavior in ApoE4 mice.

### 3.2. Cu Exposure Did Not Cause Anxiety-Like Behavior, Spatial Learning, and Memory Impairment in ApoE4 Mice

**Figure 2:**
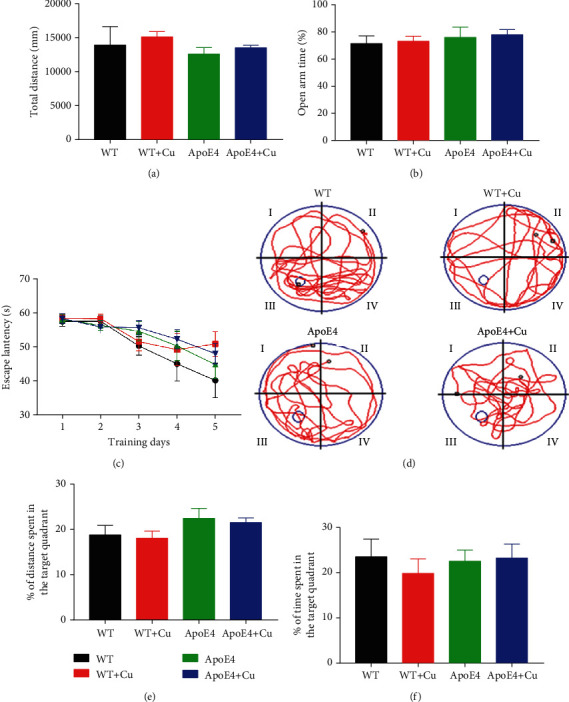
Low-dose Cu exposure did not induce anxiety-like behavior, spatial learning, and memory impairment in ApoE4 mice. (a) Total distance (mm). (b) The time spent in the open arms (%). (c) The latency 5-day training period (s). (d) The representative track diagram. (e) The distance traveled in the platform quadrant (%). (f) The time spent in the platform quadrant (%). The data was shown as mean ± SEM. *n* = 10-12 for each group.

As shown in Figure 2, the elevated plus maze test showed no difference in the total distance and open arm time among the four groups (Figures 2(a) and 2(b)), suggesting that Cu exposure did not cause anxiety-like behavior. The Morris water maze test showed that the time to find the platform was basically shorter and shorter, but there was no statistical difference (Figure 2(c)).  Figure 2(d) shows the movement trajectories of each group of mice. The travel distance and time spent in the target quadrant (Figures 2(e) and 2(f)) have no difference. These data suggested that low-dose Cu exposure had no effect on the spatial learning and memory ability of ApoE4 mice.

### 3.3. Hippocampal Proteomic Analysis

**Figure 3:**
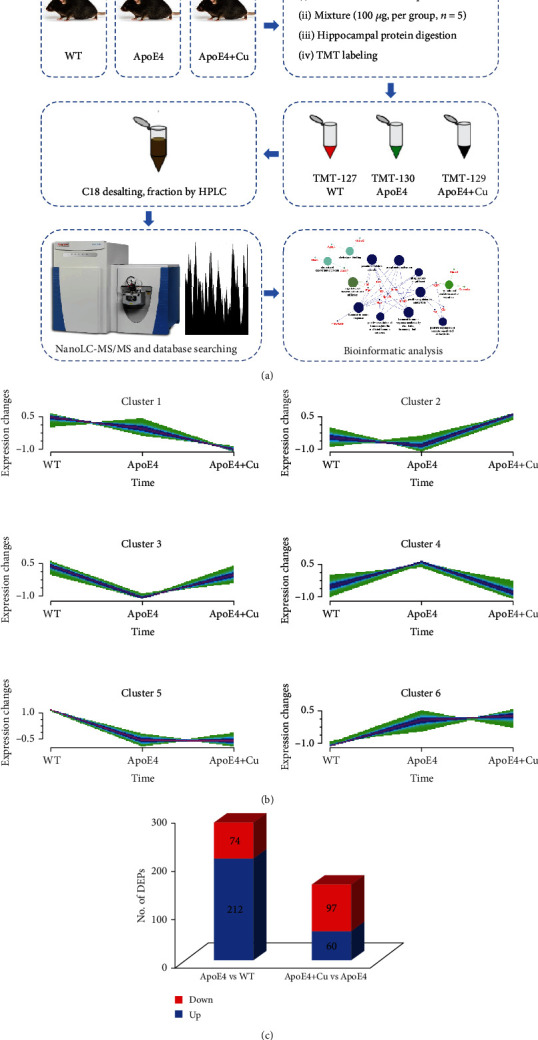
Comprehensive analysis of DEPs in the three groups of mice: (a) approaches used to evaluate hippocampal proteins; (b) clusters representing the typical expression profiles were colored accordingly to the DEPs; (c) numbers of the upregulation and downregulation of DEPs.

Following the flowchart of proteomic research, we explored the hippocampal proteome by LC-MS/MS analysis for the WT, ApoE4, and Cu-treated ApoE4 mice (Figure 3(a)). A total differentially expressed proteins (DEPs) are, respectively, divided into six categories through cluster analysis (Figure 3(b)). Cluster 1 showed a trend toward a decrease in the DEPs, while Cluster 3 trended toward an increase. Furthermore, as shown in Figure 3(c), a total of 286 proteins were identified between WT mice and ApoE4 mice. Compared to the WT group, 212 proteins were upregulated in ApoE4 mice and 74 proteins were downregulated. A total of 157 proteins were identified between ApoE4 mice and Cu-treated ApoE4 mice. Compared with ApoE4 mice, 97 and 60 proteins were upregulated and downregulated. Together, Cu exposure can cause changes in the rise or fall of hippocampal proteins. The false discovery rate (FDR) was <1%.

### 3.4. Gene Ontology Analysis for the Differentially Expressed Proteins (DEPs)

**Figure 4:**
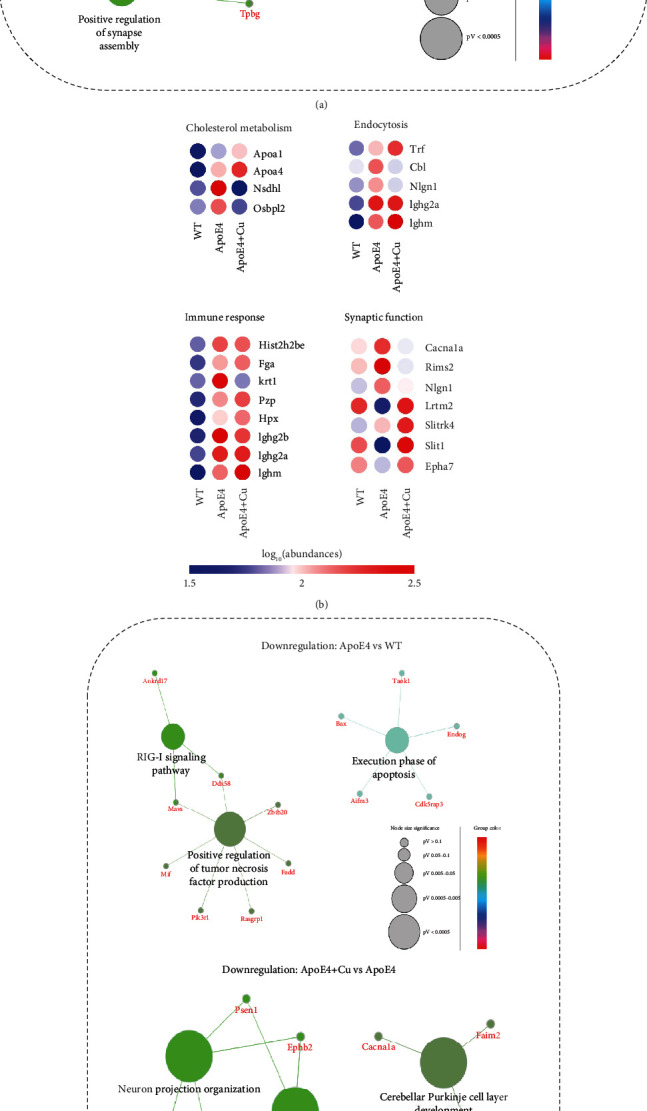
Clue GO and heat map analysis of the differentially expressed proteins. Differentially expressed proteins identified in (a) upregulations and (c) downregulations. The enriched GO terms were organized in different colors; gene names are linked to related items. Heat map analysis of DEPs localized to (b) cholesterol metabolism, endocytosis, immune response, and synaptic function and (d) synaptic function, immune response, and apoptosis. Blue color represents low abundance, and red represents high abundance.

Gene ontology analysis is used to annotate hippocampal DEPs and analyze their involvement in biological processes (Figure 4). The upregulated proteins noted that the obvious interactions were associated with cholesterol metabolism, endocytosis, immune response, and synaptic function, while the downregulated proteins were involved in synaptic function, immune response, and apoptosis (Figures 4(a)–4(d)). Interestingly, synaptic function and immune response were found in both upregulated and downregulated DEPs, indicating involvement of synaptic impairment and immune response in aggravated depression-like behavior in Cu-treated ApoE mice. By using heat map analysis, we further visualized these DEPs involved in the functional classification (Figures 4(b) and 4(d)). In addition, these DEPs from Cu-treated WT mice compared with WT mice were mainly enriched in spliceosome, mRNA processing, endocytosis, and transcription (Supplement Figure 1). These data suggested that the depression-like behavior induced by low-dose copper exposure may be related to synaptic function and immune response.

### 3.5. Enrichment Analysis of the KEGG Signaling Pathway of the Differentially Expressed Proteins

**Figure 5:**
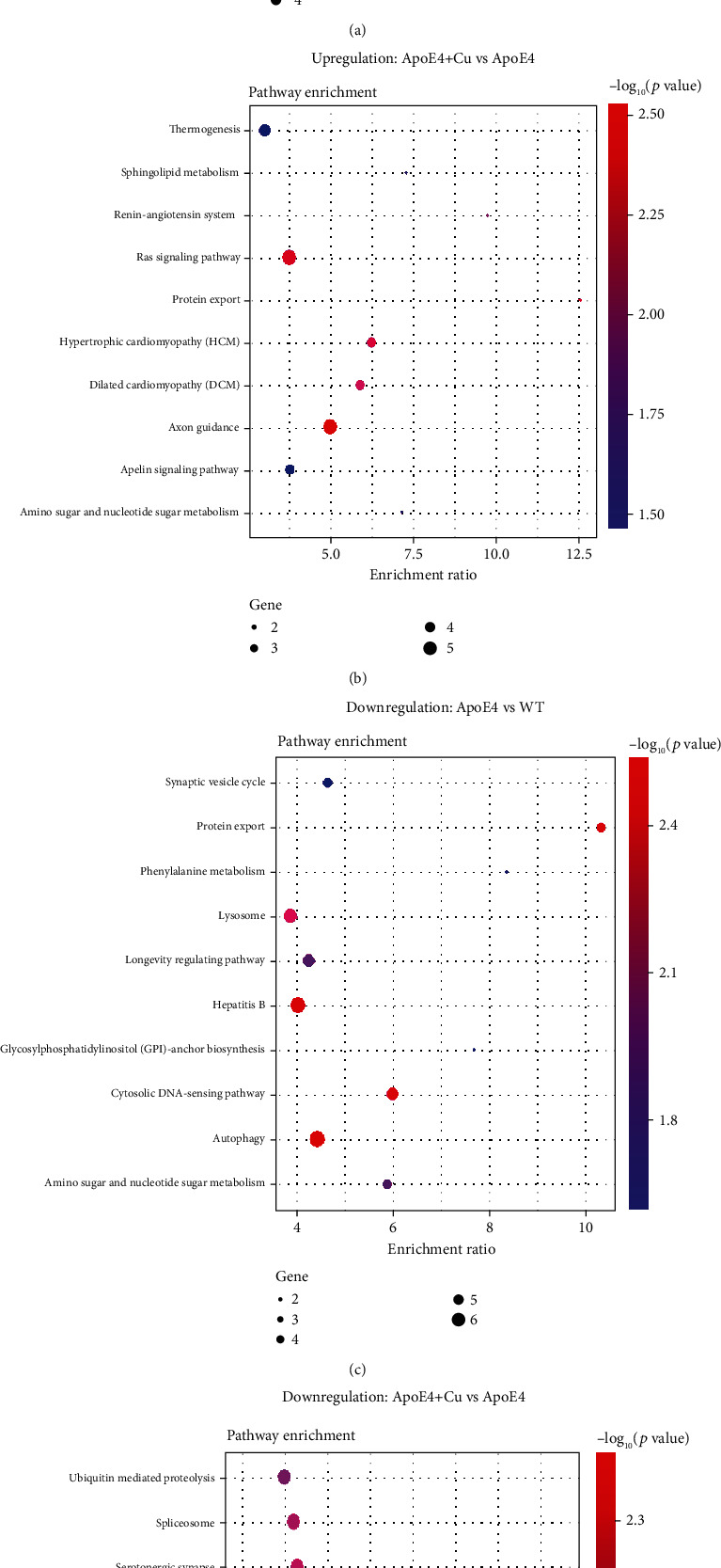
The KEGG pathway analysis for DEPs. (a, b) The enriched pathways in upregulation proteins of WT, ApoE4, and Cu-treated ApoE4 mice. (c, d) Downregulation proteins of WT, ApoE4, and Cu-treated ApoE4 mice.

**Figure 6:**
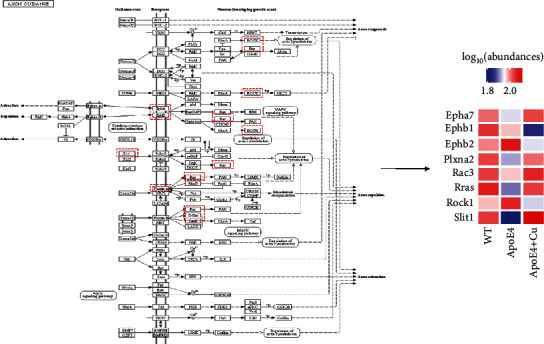
Axon guidance pathway analysis for the DEPs. Heat map displayed as nodes labeled with gene names. The red and blue boxes represent upregulation and downregulation, respectively.

Signaling pathways affected by the low-dose Cu exposure were detected by KEGG analysis, and we identified the top 10 enrichment terms (Figure 5). Compared with WT mice, the upregulated DEPs in ApoE4 mice were mainly involved in systemic lupus erythematosus, alcoholism, viral carcinogenesis, vitamin digestion and absorption, complement and coagulation cascades, fat digestion and absorption, platelet activation, cholesterol metabolism, ubiquinone and other terpenoid-quinone biosynthesis, and steroid biosynthesis (Figure 5(a)), while the downregulated DEPs in ApoE4 mice were mainly involved in autophagy, protein export, hepatitis B, cytosolic DNA sensing pathway, lysosome, longevity regulating pathway, amino sugar and nucleotide sugar metabolism, phenylalanine metabolism, synaptic vesicle cycle, glycosylphosphatidylinositol- (GPI-) anchor biosynthesis (Figure 5(c)). Likely, compared with ApoE4 mice, the upregulated DEPs in Cu-treated ApoE4 mice were mainly involved in axon guidance, Ras signaling pathway, protein export, hypertrophic cardiomyopathy (HCM), dilated cardiomyopathy (DCM), renin-angiotensin system, sphingolipid metabolism, amino sugar and nucleotide sugar metabolism, thermogenesis, and apelin signaling pathway (Figure 5(b)), while the downregulated DEPs in Cu-treated ApoE4 mice were mainly involved in GABAergic synapse, morphine addiction, nicotine addiction, serotonergic synapse, spliceosome, dopaminergic synapse, ubiquitin-mediated proteolysis, retrograde endocannabinoid signaling, long-term depression, and axon guidance (Figure 5(d)). Obviously, axon guidance was found in both upregulated and downregulated DEPs in Cu-treated ApoE4 mice compared with ApoE4 mice, suggesting that axon guidance may be an important pathway in the regulation of Cu-aggravated depression-like phenotype of ApoE4 mice. Lastly, as shown in Figure 6, some key DEPs including Epha7, Ephb1, Ephb2, Plxna2, Rac3, Rras, Rock1, and Slit1 were involved in the regulation of axon guidance.

### 3.6. Validation of Protein Expression Levels by Western Blot Analysis and the Levels of Inflammatory Cytokines

**Figure 7:**
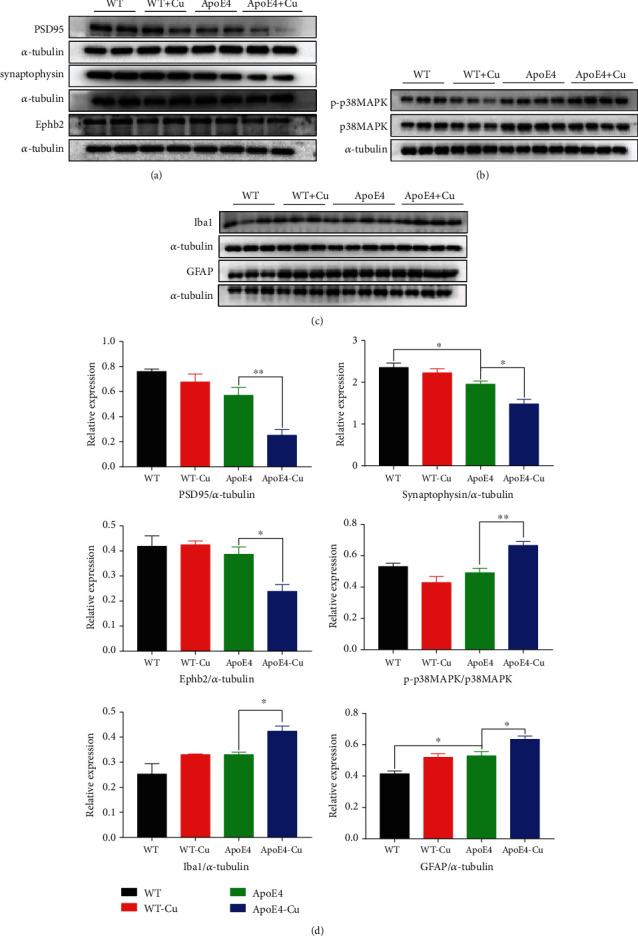
Western blot analysis validation for the changed proteins. (a, d) Quantified synaptic proteins, such as synaptophysin, PSD95, EphB2, p38 MAPK, and phospho-p38 MAPK expression, were validated. (b–d) The expression level and quantified proteins of the GFAP and Iba1. The data was shown as mean ± SEM. ^∗^*p* < 0.05, ^∗∗^*p* < 0.01. *n* = 3-4 for each group.

Western blotting analysis was used to validate the data obtained by hippocampal proteomic analysis. Seven proteins (p38 MAPK, phospho-p38 MAPK, synaptophysin, PSD95, EphB2, GFAP, and Iba1) were selected for validation (Figure 7). Western blot analysis showing that synaptophysin was downregulated in the ApoE4 mice, compared with the WT mice. PSD95, EphB2, and synaptophysin were significantly reduced in the hippocampus of Cu-treated ApoE4 mice vs. ApoE4 mice. Meanwhile, compared with ApoE4 mice, the changes of SYN1, GluR1, and GluR2 were significantly downregulated in Cu-treated ApoE4 mice, while GluR1 and GluR2 were lower in ApoE4 mice vs. WT mice (Supplement Figure 2). According to the phospho-p38 MAPK/p38 MAPK, GFAP and Iba1 showed a significant increase in Cu-treated ApoE4 mice compared with ApoE4 mice. In addition, the levels of proinflammatory factors IL-6 and TNF-*α* in ApoE4 mice were increased after low-dose copper exposure, and the levels of proinflammatory factors IL-6 were increased in ApoE4 mice vs. WT mice (Supplement Figure 3). These data suggested that an impaired synaptic function and activation of neuroinflammation were involved in depression-like behavior as we observed.

### 3.7. The Analysis of Astrocytes and Microglia in Mice

**Figure 8:**
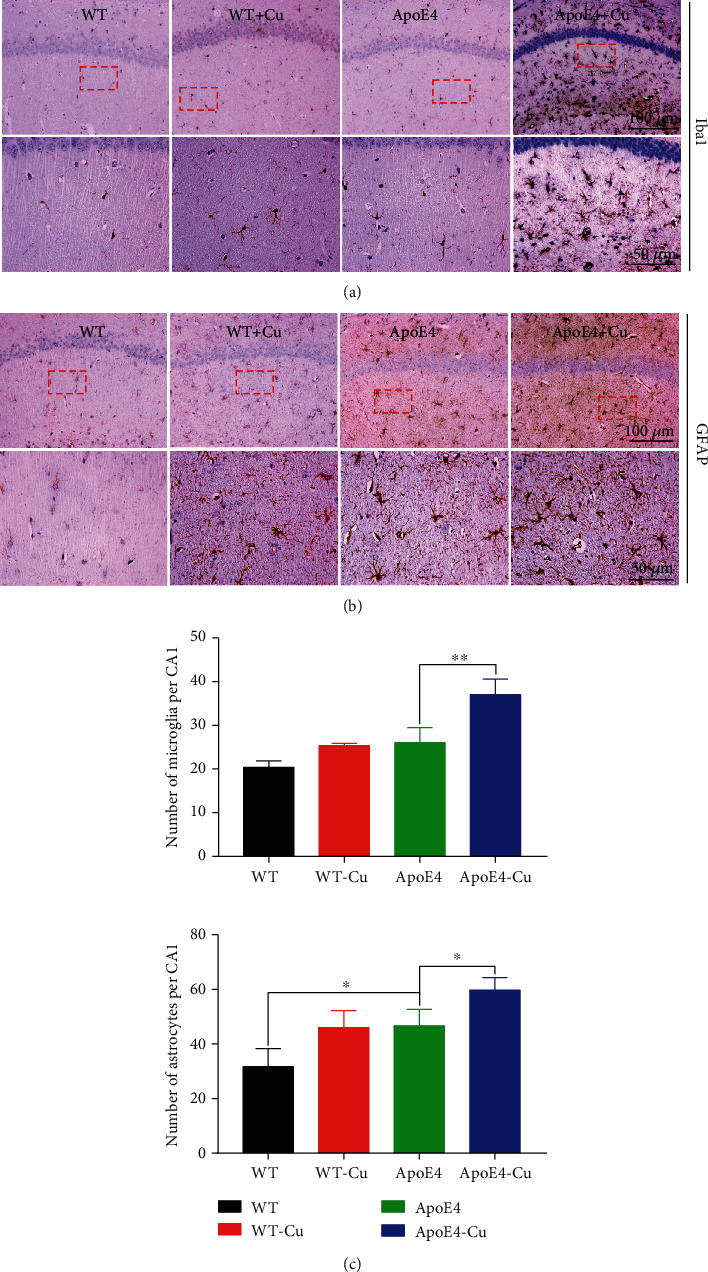
Low-dose copper exposure increased the number of astrocytes and microglia. Immunoreactivity of astroglia (GFAP) and microglia (Iba1) in the CA1 of the hippocampus. (a–c) The representative image of positive staining of GFAP (astrocytes) and Iba1 (microglia) in the CA1 hippocampus and quantitative analysis. The scale: 100 *μ*m and 50 *μ*m. The data was shown as mean ± SEM. ^∗^*p* < 0.05, ^∗∗^*p* < 0.01. *n* = 3-4 for each group.

We used immunohistochemistry to detect the expression of astrocyte (GFAP) and microglia (Iba1) in the in hippocampal tissues (Figures 8(a) and 8(b)). Astrocyte proliferation and microglial activation are two of the main features of neuroinflammation [26, 27]. The level of astrocytes was significantly increased in ApoE4 mice vs. WT mice (Figure 8(c)). Cu treatment increased the positive immunostaining of GFAP and Iba1 in ApoE4 mice, while the number of astrocytes and microglia was significantly increased (Figure 8(c)). All these results suggested that low-dose Cu exposure activates the neuroglia.

## 4. Discussion

The role of Cu and ApoE4 in depression is well established. Multiple studies have focused on the effect of Cu exposure or ApoE4 on depression; however, the interactions of Cu exposure and ApoE4 on depression are little reported. We found that ApoE4 mice showed depression-like behavior, which was further aggravated by Cu exposure. Our results revealed changes in synaptic proteins and neuroinflammation, which can explain the factors that may lead to depression.

Our findings indicated that low-dose Cu exposure exacerbates depressive actions in the ApoE4 mice, showing the potential underlying causes of depression. ApoE is a polymorph protein involved in the transformation and metabolism of lipoproteins, and its gene can regulate many biological functions that have been existed as a risk factor in mental diseases, such as depression [28]. Additionally, the disturbance of Cu metabolism can cause neuropsychiatric symptoms, such as depression [29]. Therefore, ApoE4 mice showed aggravation of depression-like behavior under Cu exposure. However, the mice showed no cognitive impairment on the Morris water maze test, possibly related to age.

In our study, we successfully identified multiple differentially expressed proteins in three groups of mice, in which proteomic changes associated with synaptic function were prominent. Synapses are the functional connections between neurons and the key sites of information transmission [30]. Many mental and neurological diseases are associated with the destruction of the number and shape of synapses [31]. In our findings, ApoE4 mice were exposed to Cu for 4 months, which caused Cu ions to accumulate in the brain and interfere with neuronal activity. The degeneration of nerve cells induced by oxidative stress caused by copper will lead to the dysfunction of synapses [6, 32]. As for the effects of ApoE on the nervous system, it has been found that it may involve several pathways, such as destruction of synaptic plasticity, neuroinflammation, abnormal lipid, and glucose metabolism [33]. Moreover, several studies showed that depression is associated with a decrease number of synapses and synaptic dysfunction in brain regions that control emotion and cognition. And researchers used electron microscopy to provide direct evidence in a group of depressed subjects [34–36]. Preclinical studies have also further reported low alterations of the density of synapses in depression models, especially in the prefrontal cortex and hippocampus [34, 37]. This result was in agreement with our proteomic work analysis, indicating that synaptic dysfunction is closely associated with depression. Notably, according to proteomic analysis, the expression of EphB2 was significantly downregulated in the Cu-treated ApoE4 mice. EphB plays an important role in early synaptic formation, which is involved in the regulation of synaptic efficacy in mature neurons to localize synapses and regulate glutamate receptor function [38, 39]. Ephrins and Eph receptors are widely distributed throughout the nervous system, such as EphA4, EphB1, EphB2, and EphB3. Their downregulation resulted in decreased synaptic function, mood changes, and cognitive impairments such as depression and anxiety [40–42]. Another possible mechanism revealed the role of p38 MAPK in neuronal plasticity and synaptic regulation and suggested that the activation of the p38 MAPK signaling pathway damages synaptic function, for which we have also validated the p38 MAPK pathway [43]. Interestingly, recent studies have uncovered that the MAPK signaling pathway is involved in the occurrence of depression, and blocking the MAPK signaling pathway can alleviate depression. This phenomenon may be associated with synaptic dysfunction caused by activation of p38-MAPK-mediated signal cascade [44, 45]. By proteomics and western blotting, we found that synaptic dysfunction may be a possible mechanism for the deterioration of depression-like behavior in Cu-treated ApoE4 mice.

The next question is to answer how the immune response contributes to the development of depressive symptoms. Multiple increasing evidences suggested that the dysfunction of immune function is a main feature of neuropsychiatric disorders, in particular in neurodevelopment [46]. Immune signaling molecules played key roles in neurodevelopment, and many of which are glial sources (such as microglia and astrocytes). Among them, microglia are innate immune cells in the brain, while astrocytes are the key participants of central nervous system immune responses [47, 48]. It has been reported that the activation of microglia was obviously increased in depression patients, and nerve damage induced by microglia is considered to be an important mechanism of depression [49–51]. In addition, some antidepressants, such as minocycline, can play a protective role by inhibiting the activation of microglia, oxidative stress, and inflammation [52, 53]. Astrocytes are involved in synapse formation, maturation, function, and plasticity. Mounting evidence suggested that astrocytes play a pivotal role in central nervous system diseases, such as neuropsychiatric diseases [54, 55]. These findings suggested that the occurrence of depression is associated with activation of the glia. Besides, glial cells are not only important participants in immune response but also the basis of neuroinflammation. It was worth noting that neuroinflammation is the complex innate immune response of neural tissue to inhibit infection and remove pathogens, cell fragments, and misfolded proteins [56, 57]. Thus, we examined the expression of GFAP and Iba1 in the hippocampus of mice by western blotting and immunohistochemistry, and their expressions were confirmed to be upregulated. More interestingly, glial cells also affect major aspects of synaptic development, plasticity, and function [58]. Moreover, in the presence of ApoE, glial cell proliferation was increased, thereby promoting an inflammatory response, while immune response is generated by the central nervous system injury caused by excessive Cu. Therefore, it is clear that the factors of depression depend on the complex process of synapses, immune response, and neuroinflammation, which is also a promising point for our future research on depression.

## 5. Conclusion

In sum, our study demonstrated that Cu exacerbated depression-like behavior in ApoE4 mice and provided the hippocampal proteomic characterization. The major contribution of this study is providing strong support for the involvement of synaptic function in the development of depression. Additionally, impaired immune response and overactivated neuroinflammation are also important factors in the development of depression.

## Data Availability

All the proteomic data were deposited with the ProteomeXchange Consortium via the PRIDE partner repository with the dataset identifier PXD022422.

## Supplementary Materials

Supplementary Materials Supplementary Figure 1: clue GO analysis of the differentially expressed proteins. Differentially expressed proteins identified in upregulations and downregulations of Cu-treated WT mice vs. WT mice. The enriched GO terms were organized in different colors; gene names are linked to related items. Supplementary Figure 2: low-dose copper exposure aggravated synaptic impairment in ApoE4 mice. The expression of GluR1, GluR2, NMDA-2A, NMDA-2B, and SYN1 was detected by western blotting and quantitative analysis. The data was shown as mean ± SEM. ^∗^*p* < 0.05, ^∗∗^*p* < 0.01, and ^∗∗∗^*p* < 0.001. *n* = 4 for each group. Supplementary Figure 3: low-dose copper exposure aggravated neuroinflammation. (A-C) IL-6, TNF-*α*, and IL-10 ELISA. Cu significantly increased the concentrations of IL-6 and TNF-*α* in ApoE4 mice, but it has no effect on IL-10.The data was shown as mean ± SEM. ^∗^*p* < 0.05. *n* = 4 for each group.
