# Supplementary figures and images for: Low-Dose Copper Exposure Exacerbates Depression-Like Behavior in ApoE4 Transgenic Mice
Source: Oxid Med Cell Longev. 2021 Mar 25;2021:6634181. doi: 10.1155/2021/6634181 (PMC8018851; doi:10.1155/2021/6634181)

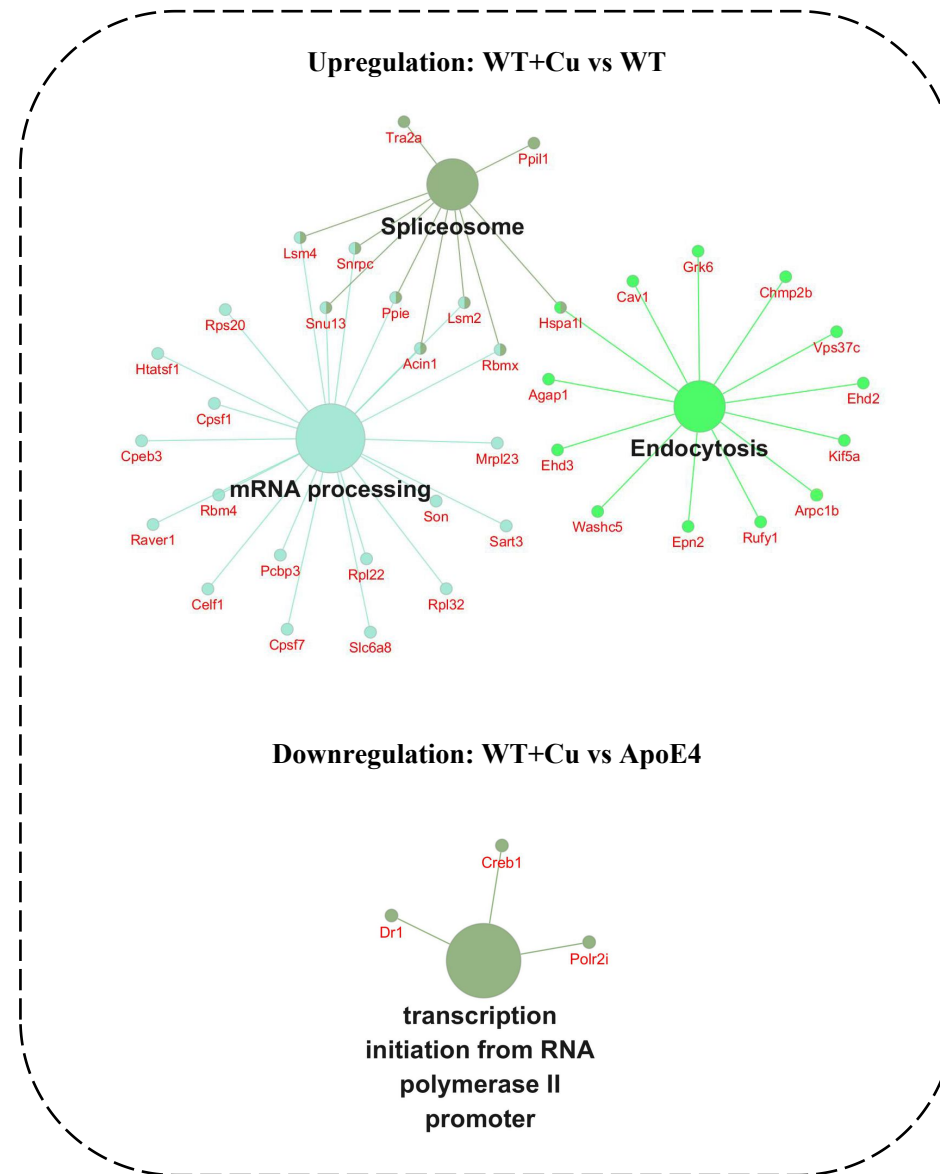

Supplementary Figure 1

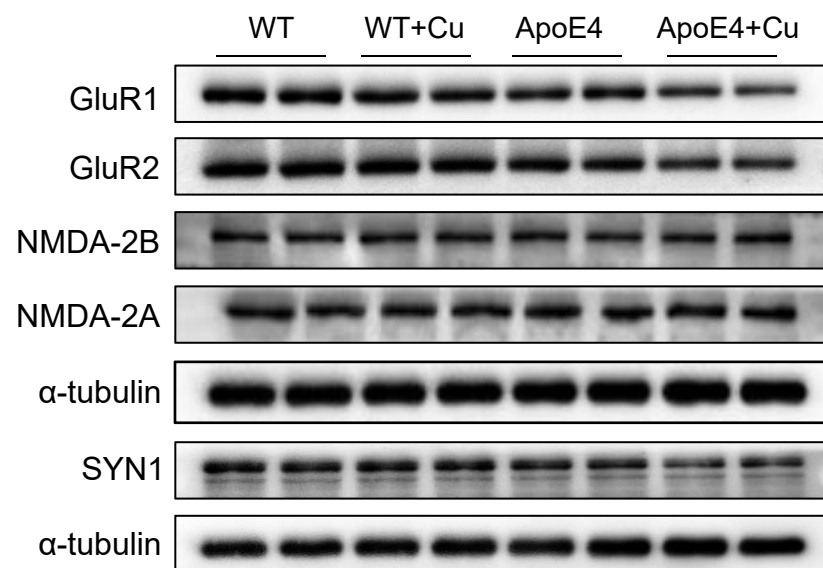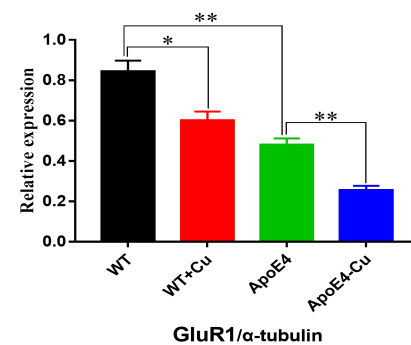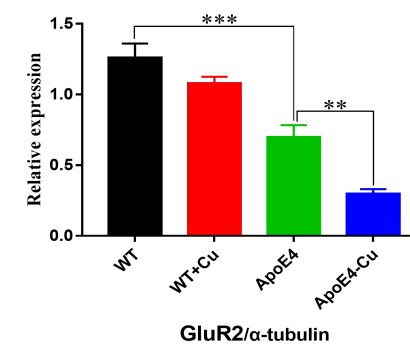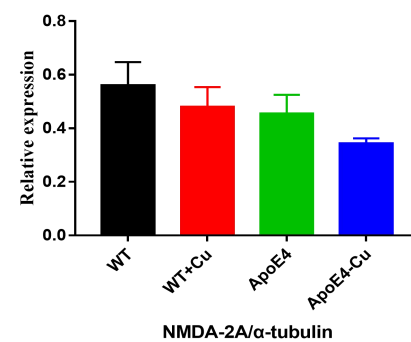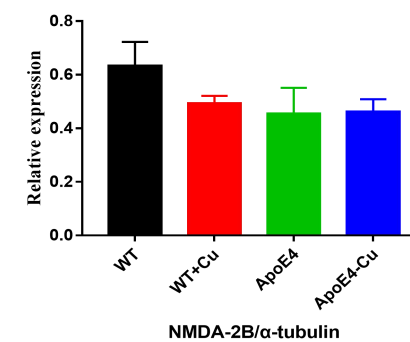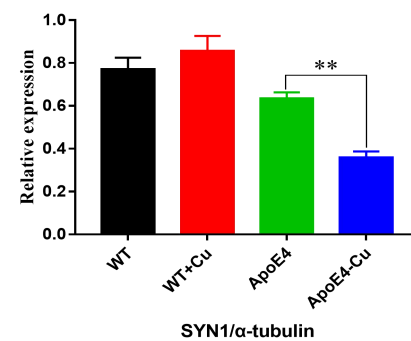

Supplementary Figure 2

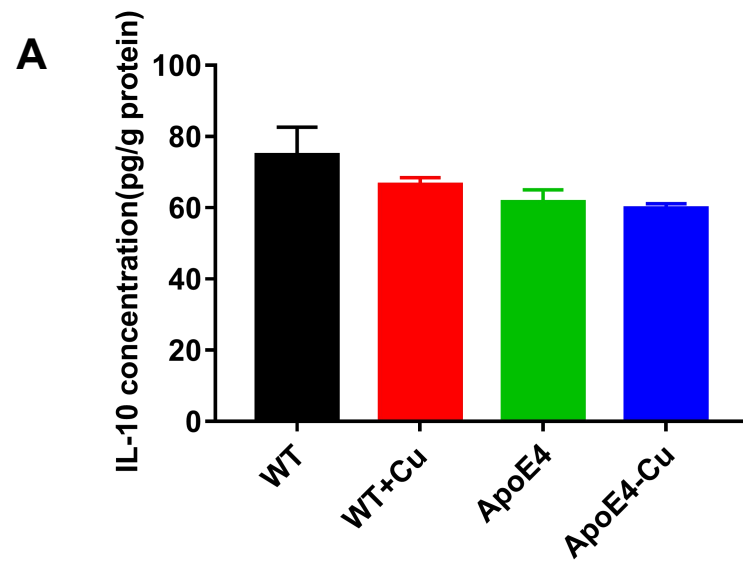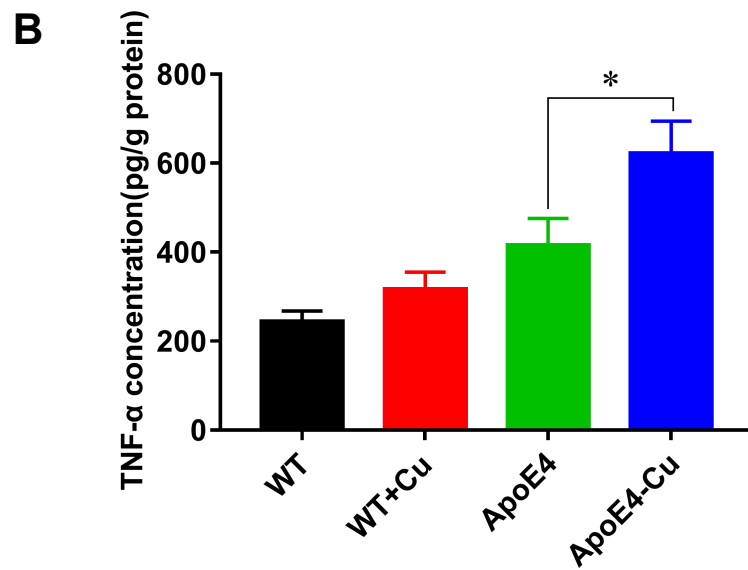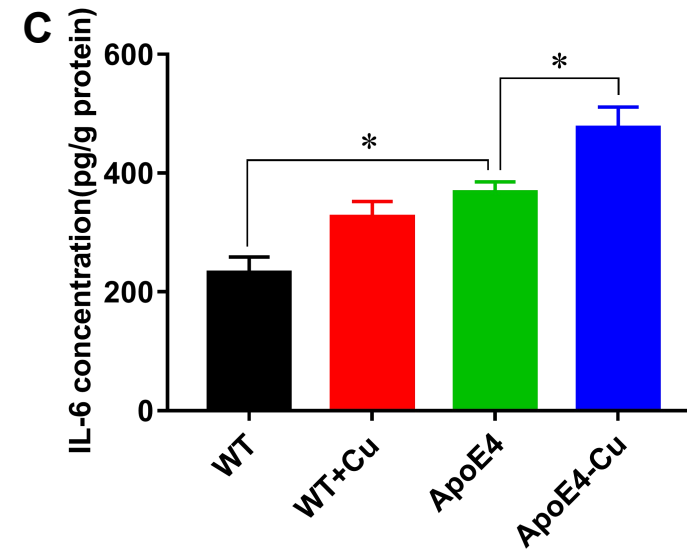

Supplement: Supplementary Materials — Supplementary Figure 1: clue GO analysis of the differentially expressed proteins. Differentially expressed proteins identified in upregulations and downregulations of Cu-treated WT mice vs. WT mice. The enriched GO terms were organized in different colors; gene names are linked to related items. Supplementary Figure 2: low-dose copper exposure aggravated synaptic impairment in ApoE4 mice. The expression of GluR1, GluR2, NMDA-2A, NMDA-2B, and SYN1 was detected by western blotting and quantitative analysis. The data was shown as mean ± SEM. ∗p < 0.05, ∗∗p < 0.01, and ∗∗∗p < 0.001. n = 4 for each group. Supplementary Figure 3: low-dose copper exposure aggravated neuroinflammation. (A-C) IL-6, TNF-α, and IL-10 ELISA. Cu significantly increased the concentrations of IL-6 and TNF-α in ApoE4 mice, but it has no effect on IL-10.The data was shown as mean ± SEM. ∗p < 0.05. n = 4 for each group. [file 6634181.f1.pdf]
